# Hotspot gene mutations and treatment response in myelodysplastic syndromes (MDS): predictive biomarkers and targeted strategies

**DOI:** 10.3389/fmed.2025.1712877

**Published:** 2026-01-05

**Authors:** Rui Zhang, Wei Kou, Tao Wu, Rui Zhou

**Affiliations:** 1 Department of Hematology, Center for Hematologic Diseases of Chinese PLA, The 940th Hospital of Joint Logistics Support force of Chinese People’s Liberation Army (Lanzhou Military Command General Hospital), Lanzhou, Gansu, China; 2 Medical Department, Northwest Minzu University, Lanzhou, Gansu, China

**Keywords:** myelodysplastic syndromes, hypomethylating agents, mutation, chemotherapy, targeted therapy

## Abstract

The myelodysplastic syndromes (MDS) are clonal hematopoietic stem cell disorders characterized by cytopenia and a high risk of transformation to acute myeloid leukemia. In recent years, next-generation sequencing (NGS) has revealed common hotspot gene mutations in MDS, which are not only involved in disease progression, but also affect the responsiveness of different therapeutic strategies. Current research has revealed that *ASXL1* mutations in MDS predict demethylating agents (HMAs) resistance, the combination of HMAs and Venetoclax (VEN) achieved an ORR of 87%. DNMT*3A R882* mutations induce decitabine sensitivity via hemi-methylated enhancer trapping, and *TET2* mutations enhance HMAs efficacy only in *ASXL1* wild-type contexts (ORR 62.1% vs. co-mutated 19%). *RUNX1* aberrations reduce chemotherapy responses (18.9% ORR in high-risk MDS) through DNA repair impairment, while *BCOR/EZH2* loss drives cytarabine resistance. *TP53* multi-hit lesions correlate with poor survival (OS <12 months) but respond to eprenetapopt-azacitidine (ORR 73%), and *IDH1/2* inhibitors achieve durable remissions (ivosidenib ORR 83.3%, mOS 35.7 months). In this paper, we systematically illustrate the correlation between key gene mutations and the response to HMAs, chemotherapy and targeted therapies in MDS patients. This article summarizes the current evidence on gene mutations as predictive biomarkers and discusses the direction of individualized therapy.

## 1 Introduction

According to previous studies, myelodysplastic syndromes (MDS) are a heterogeneous group of clonal myeloid disorders originating from hematopoietic stem cells, characterized by ineffective hematopoiesis, refractory cytopenias, and an elevated risk of progression to acute myeloid leukemia (AML), and are more common in older males and individuals previously exposed to cytotoxic therapy (1, 2). The current goal of MDS treatment is to improve hematopoietic function and quality of life, while the goal of treatment for higher-risk MDS (HR-MDS) patients is to delay disease progression, prolong survival, and pursue a cure (3–5).

Current therapeutic approaches for MDS, including supportive care (such as blood component transfusions, infection prevention and treatment, and iron chelation therapy), hematopoietic stimulation, biological response modifiers (e.g., lenalidomide and thalidomide), hypomethylating agents (HMAs) [e.g., Azacitidine (AZA) and Decitabine (DEC)], combination chemotherapy and allogeneic hematopoietic stem cell transplantation (allo-HSCT), are limited by suboptimal response rates and poor long-term outcomes (1, 2). For example, supportive therapy can only provide symptomatic relief and is not curative. In addition, long-term blood transfusions can result in iron overload (6, 7). The overall response rate (ORR) to HMAs in low-risk MDS was approximately 40–50%, while in high-risk patients, it dropped below 20–30% (8–10).

Moreover, conventional prognostic systems demonstrate insufficient integration of genomic profiling, which consequently manifests as heterogeneity in therapeutic outcomes (1, 2, 11, 12). Technological advancements in genomic profiling methodologies, illustrated by the 2022 IPSS-M refinement incorporating somatic mutation analysis, have demonstrate the predictive value of mutations in genes such as *Additional Sex Combs Like 1(ASXL1)*, *Ten-Eleven Translocation 2 (TET2)*, and *Tumor Protein P53 (TP53)* with respect to treatment refractoriness (13) (Table 1).

**Table 1:** Comprehensive information on MDS-related mutated genes (2, 14–16).

Mutated gene	Function	Location	Prevalence	Prognostic implications
ASXL1	Involved in chromatin remodeling and gene expression regulation	20q11	11% ~ 21%	Adverse prognosis, associated with disease progression and shortened survival
TET2	Regulates DNA demethylation and maintains gene expression balance	4q24	21%	Relatively favorable prognosis, associated with increased sensitivity to HMAs
DNMT3A	Involved in DNA methylation and gene silencing	2p23.3	5% ~ 12%	Prognostic significance remains unclear; some studies suggest possible association with treatment response
RUNX1	Involved in the maintenance and differentiation of hematopoietic stem cells	21q22.12	5% ~ 10%	Adverse prognosis, associated with poor response to chemotherapy
BCOR	Involved in transcriptional regulation and chromatin modification	Xp11.4	2% ~ 5%	Adverse prognosis, associated with chemoresistance
EZH2	Mediates histone methylation and regulates gene silencing	7q36	6%	Adverse prognosis, associated with disease progression
TP53	Regulates cell cycle, DNA repair, and apoptosis	17q13.1	5% ~ 10%	Extremely poor prognosis, associatedwith rapiddisease progressionand significantly shortened survival
IDH1/IDH2	Involved in cell metabolism and oxidative stress regulation	2q33/15q26	0.6 ~ 3.5/2.1% ~ 4.0%	Prognosis depends on mutation type; some mutations are associated with a better prognosis

These mutations not only drive disease pathogenesis but also serve as biomarkers for personalized treatment selection. This review provides an overview of the role of hotspot gene mutations in MDS, focusing on their predictive value as biomarkers and their potential in guiding targeted therapeutic strategies to address the limitations of current therapies and improve patient outcomes.

## 2 Mutations predicting response to hypomethylating agents

DNA methylation alterations are commonly observed in AML and MDS. Previous studies have shown that, compared to somatic mutations, there is a significantly stronger association between methylation changes and the risk of pre-AML/MDS in individuals. This suggested that methylation changes could serve as one of the biomarkers for pre-AML/MDS screening (14).

HMAs such as AZA and DEC have revolutionized therapeutic strategies for MDS, but there is heterogeneity in clinical response and the certainly development of secondary resistance (2). New research evidence suggests that specific gene mutations influence HMA efficacy through epigenetic reprogramming and clonal dynamics.

### 2.1 *ASXL1* mutations: resistance to HMAs via epigenetic dysregulation

In patients with MDS, the frequency of *ASXL1* mutations is approximately 11% ~ 21%. *ASXL1* is closely associated with treatment failure of HMAs in 40% of MDS patients (15). It has been found that *ASXL1* mutations disrupt the process of neutrophil differentiation, leaving neutrophils at an immature stage (16). The reduced number and abnormal function of such immature neutrophils are typical of the abnormal neutrophil development in MDS (16). A retrospective study found that in MDS patients with ASXL1 mutations, the combination of HMAs and Venetoclax (VEN) achieved an ORR of 87%, a complete remission rate (CRR) of 44%, compared to 32% ORR and 8% CRR with HMAs alone (17). Studies have shown that *ASXL1* mutations, which cause the loss of function of *the Polycomb repressive complex 2 (PRC2)* and dysregulate gene expression—particularly overexpressing *HOXA and BCL2*—render cells more sensitive to VEN and AZA (18–20). Additionally, ASXL1 deficient cells, which exhibit abnormal cell-cycle checkpoints and DNA repair mechanisms, upregulate *BIRC5* (15). The overexpression of *BIRC5* enhances resistance to apoptosis, leading to DEC resistance. Thus, while *ASXL1* mutations can increase sensitivity to certain HMAs and targeted drugs, they can also cause resistance to other HMAs by altering cell-cycle regulation, apoptosis, and epigenetic control. The mutation status of *ASXL1* serves as a key biomarker for predicting treatment responses and guiding clinical decisions (18, 19).

### 2.2 *TET2* mutations: enhanced sensitivity through methylation reversal

*TET2* belongs to the Fe(II)/*α*-ketoglutarate-dependent dioxygenase family. Its function is to sequentially oxidize 5-methylcytosine (5mC) to generate 5-hydroxymethylcytosine (5hmC), 5-formylcytosine (5fC), and 5-carboxylcytosine (5caC), thereby initiating active DNA demethylation. This process is crucial for maintaining low methylation states at enhancers and genomic stability (21, 22). MDS-associated frameshift, missense, or splice site mutations predominantly occur within its catalytic domain (HHXDXnH motif) and C-terminal CXXC domain, resulting in a significant 60–90% reduction in 5mC catalytic efficiency. This, in turn, causes a genome-wide decrease in 5hmC levels by 30–50% (21–23). This catalytic defect permits *DNMT3A* and other DNA methyltransferases like *DNMT1* to re-establish 5mC on CpG-dense enhancers (CpG > 70%), forming anomalous hypermethylated regions of approximately 1.5–3 kb (termed “methylation canyons”). These canyons directly block binding sites for key transcription factors like *RUNX1*, *ATF3*, and *SPI1*, while recruiting MBD3–NuRD complexes to reinforce transcriptional silencing (23). Concurrently, *TET2* deficiency disrupts its physical interactions with NIPBL and NIPBL loading factors, reducing enhancer-promoter chromatin loop strength by approximately 40% (22). This dual disruption of hydroxymethylation status and three-dimensional chromatin architecture results in persistent hypermethylation of myeloid differentiation genes (*CEBPA*, *CSF1R*) and tumor suppressor loci, while transcription of self-renewal regulators (*MEIS1*, *EVI1*) is upregulated, ultimately driving abnormal HSC clonal dominance (24, 25). Furthermore, 5hmC depletion also affects the lymphoid lineage, impairing NK cell regulation of KIR and perforin promoters, promoting immune evasion, and accelerating MDS progression (26–28). Consequently, *TET2* mutations emerge as a core driver in MDS pathogenesis and a potential therapeutic biomarker (24, 26–28).

Liu et al. (29) found that the mutation frequency of *TET2* was 21% in 228 newly diagnosed Chinese MDS patients and 15% in 184 treated MDS patients by Next-Generation Sequencing (NGS), with a significantly higher mutation frequency in patients over 60 years old. This was consistent with the results of a retrospective study by Du et al. (30). Several studies have shown that the frequency of TET2 mutations in Asian MDS patients is significantly lower than that in Western populations, at 17.5 and 28.9%, respectively, and that the frequency of *TET2* mutations remains stable during disease progression to AML (31, 32). In Chinese MDS patients, *TET2* mutations are more common and age-related, which may be a key factor in the pathogenesis of MDS and its transition to AML, and may serve as a potential marker for early disease onset (30, 33, 34). In addition, the mutation rates of *SF3B1*, *TET2*, *ASXL1* and Serine and *Arginine Rich Splicing Factor 2 (SRSF2)* in Chinese MDS patients were lower than those in Western patients, whereas the mutation rates of *TP53* and *RUNX1* were relatively high, which may be related to the ethnic background (31, 33).

Numerous studies have demonstrated that HMAs play a therapeutic role by inhibiting DNA methyltransferase activity, reversing the aberrant DNA methylation pattern caused by *TET2* mutations, and restoring normal gene expression (35). In addition, HMAs were able to upregulate the expression of genes related to inflammation and apoptosis and enhance the anti-leukemic function of Natural Killer Cells (NK cells). Meanwhile, *TET2* mutations may synergized with other gene mutations [e.g., *DNA Methyltransferase 3A (DNMT3A)*, *Isocitrate Dehydrogenase 2(IDH2)*, etc.] to affect the response of MDS patients to hypomethylating agents. HMAs enhanced the therapeutic effect by reversing the synergistic effect of these mutations through multi-targeted action (36–38). Dame’s et al. (39) study found, in the 121 patients treated with HMA, the response rate in patients with *TET2* mutations was 40%, comparable to wild-type *TET2* patients (41%). Anthony et al. (40) retrospective study found that the *TET2* mutation/*ASXL1* wild-type genotype was the strongest predictor of response to HMA treatment (ORR of 62.1%, CRR of 34.5%). Although HMAs had emerged as a first-line treatment for MDS patients who are not candidates for intensive chemotherapy, its efficacy was poor in the majority of patients. Response to these agents in patients with MDS usually occured after the 4th or 6th cycle of treatment and was of short duration. In addition, *TET2* mutations were not good biomarkers for predicting patient response to HMAs (41–45). Consequently, *TET2* alone is insufficient for response prediction; combined *TET2/ASXL1* genotyping defines a biomarker-positive subgroup that achieves durable remission, whereas dual-mutant cases should be considered for upfront transplantation or combination trials.

### 2.3 Emerging insights into *DNMT3A* mutations

Acting as a DNA methyltransferase, *DNMT3A* mainly catalyzes *de novo* DNA methylation on the cytosine residues of CpG islands. Previous findings have demonstrated that *DNMT3A* mutation variants could serve as potential biomarkers for HMA response in MDS and AML patients, with implications for risk stratification and therapeutic choices (36, 46). During early hematopoiesis, the de novo DNA methyltransferase *DNMT3A* establishes initial 5-methylcytosine (5-mC) patterns specifically at CG dinucleotides within CpG-island shores and enhancers. This methylation locks key hematopoietic regulators (e.g., *RUNX1*, *SPI1*) in a transcriptionally primed but repressed state (36). Critically, *DNMT3A* mutations clustering at the R882 residue within its catalytic domain (Pro-Cys-Pro loop) induce profound functional consequences. This hotspot mutation destabilizes the active *DNMT3A-DNMT3L* tetrameric complex, reducing catalytic efficiency (K_cat_/K_m_) for S-adenosylmethionine while paradoxically increasing enzyme processivity (46). The resulting “half-methylated” (hemi-5mC) CpG states at enhancers serve as pathognomonic epigenetic lesions reflective of this dysfunctional hyperprocessivity. These hemi-methylated sites exhibit exceptionally high affinity for decitabine-triphosphate (DAC-TP). Incorporation of DAC-TP forms a covalent *DNMT3A–DNA* adduct, ultimately triggering proteasomal degradation of the mutant *DNMT3A* protein and inducing localized erasure of aberrant enhancer methylation within two HMA treatment cycles (47). The consequent re-opening of critical enhancers (e.g., at *RUNX1* and *SPI1* loci) restores myeloid differentiation transcript programs, explaining the improved clinical response observed in patients harboring R882-mutant clones (46, 48–50).

In MDS cohorts, *DNMT3A* mutation was detected in 12.6% of cases and was associated with differential responses to HMAs (47). *DNMT3A R882* mutation puts MDS at higher risk for disease and AML transformation. In the follow-up of MDS patients treated with HMAs (over 70 months of follow-up), the median Progression-Free Survival (PFS) in the *DNMT3A R882* mutation group was 20.3 months, which was significantly lower than the 50 months in the non-mutated group (48).

In conclusion, *DNMT3A* can mediate DNA methylation from the beginning, and once it is mutated, it will lead to the disturbance of DNA methylation pattern and affect the gene expression. HMAs can inhibit the activity of *DNMT3A* and promote the re-expression of abnormally methylated genes to play an anti-tumor role. For cells with *DNMT3A R882H* mutation, genes related to hematopoietic differentiation are more likely to be demethylated and re-expressed under the treatment of HMAs, which promotes the normal differentiation of hematopoietic cells, improves the hematopoietic function of the patients, and strengthens the patient’s response to the treatment of HMAs (46, 49, 50).

Thus, the *DNMT3A R882* mutation functions not merely as an adverse risk factor, but as a druggable “molecular switch”: It generates a quantifiable epigenetic vulnerability to HMAs by trapping mutant protein at hypomethylated enhancers, thereby creating a distinct therapeutic window. Precision therapy strategies require prospective co-mutation profiling—particularly *ASXL1* and *IDH2* status—combined with early integration of adjunct therapies targeting residual repressive complexes (e.g., PRC2 inhibitors like CPI-1205) or modulating methyl availability to maximize exploitation of this enzymatic vulnerability. DNMT*3A R882* mutations epitomize a therapeutically actionable paradox: By pathologically enhancing enzyme processivity to create hemi-methylated enhancer “traps”, they convert an adverse driver into a druggable vulnerability-one whose clinical exploitation necessitates precision co-mutation mapping to counter compensatory epigenetic resilience.

## 3 Mutations predicting response to chemotherapy

In HR-MDS patients, those with an increased percentage of primitive cells have a worse prognosis. Chemotherapy, as one of the important treatments for non-HSCT HR-MDS patients, is often chosen as a treatment regimen for AML-like conditions, and standard 3 + 7 induction regimens for AML and pre-stimulation regimens are in clinical using. Pre-excitation regimens have been widely used in China for HR-MDS patients, consisting of a combination of G-CSF and Aclacinomycin (ACM), homoharringtonine (HHT), or Idarubicin (IDA) on top of low-dose cytarabine. The pre-stimulation regimen has been shown to achieve CRR of 40 to 60% in HR-MDS, and is better tolerated by older or less able-bodied patients than conventional AML chemotherapy regimens. In addition, pre-stimulation regimens have the potential to be used in combination with demethylating agents (1, 2, 9). However, the remarkable heterogeneity of patient responses to chemotherapy highlights the critical need to identify reliable biomarkers capable of predicting treatment efficacy and elucidating potential resistance mechanisms. Recent advances in genomic profiling have demonstrated that specific gene mutations play a crucial role in modulating chemosensitivity and influencing clinical outcomes in MDS.

*RUNX1* is involved in the transcriptional regulation of multiple processes involved in hematopoietic cell growth, differentiation, apoptosis, and the prevention of malignant transformation. Abnormalities in *RUNX1* affect these fine regulatory mechanisms, leading to the onset and progression of MDS (32). *RUNX1* mutations are detected in 5 to 10% of patients upon initial diagnosis of MDS (2, 51). *RUNX1*, located on chromosome 21q22.12, is a key regulator of myeloid differentiation and strongly drives leukemic transformation (52). Noorwati et al. (53) by a systematic review and meta-analysis found that there was a significant association between *RUNX1* mutations and increased risk of AML transformation. The progression of MDS to AML occurs in a “two-hit” model, with *RUNX1* mutations involved in the first-hit (sequential genetic alterations in cellular differentiation genes) (54). Notably, the clinical significance of *RUNX1* mutations does not exist in isolation, but is profoundly influenced by the spectrum of co-mutations. For example, co-mutations in *RUNX1* with *ASXL1* have been demonstrated by multiple studies to be associated with poorer prognosis and shorter duration of response (DOR) (55).

Studies have shown that patients with *RUNX1* mutations respond poorly to treatment with single HMAs and have worse response and shorter survival when *RUNX1* is co-mutated with *ASXL1* (56). Wang et al. (57) retrospectively analyzed 103 patients with high-risk MDS and found that the rate of *RUNX1* mutation was 18.9%, and that after treatment with VEN in combination with HMAs, the 12-month median follow-up and composite CRR were 65 and 52%, respectively, in a median of 13.5 months, month overall survival and composite CRR were 65.8 and 52.9%, respectively. MDS patients with *RUNX1* mutations respond poorly to chemotherapeutic regimens, and the mechanism may involve multiple aspects affecting cell proliferation and differentiation, DNA damage and repair defects, apoptotic processes, and signaling pathways (58, 59). Khan et al. (60) showed that patients with *RUNX1-mutated* AML who received intensive chemotherapy had poorer outcomes. *RUNX1* mutations are not only a key molecular feature of MDS, but also profoundly influence the biological behavior of the disease and the response to chemotherapy. Their disruption of the hematopoietic hierarchy and complex interactions with other mutations combine to shape the transient nature of the response to chemotherapy (61). Martijn et al. (51) also found that in patients with familial thrombocytopenia with myeloid malignancy-associated AML, ORR for chemotherapy was 70 to 80%, and the 5-year overall survival (OS) was 50.4%. In addition, *RUNX1* mutations have shown potential application in the monitoring of microscopic residual disease (MRD). Preliminary studies have shown that persistent detection of *RUNX1* mutations after chemotherapy is associated with a higher risk of relapse, suggesting that they may serve as molecular markers for predicting hematologic relapse. The inclusion of *RUNX1* mutations in the MRD assessment system could help to identify high-risk patients who require more aggressive consolidation or close monitoring (62).

Mutations in some genes (e.g., *BCOR, EZH2*) have been shown to be associated with resistance to chemotherapy in patients with MDS. Mutations in the *BCOR* gene, which is more common in MDS, may lead to decreased cellular sensitivity to chemotherapy. Mutations in the *EZH2* gene have also been associated with resistance to chemotherapy. Loss-of-function mutations in *EZH2* can cause leukemia cells to become resistant to chemotherapy, resulting in poor treatment outcomes. In one study, mutations in the *BCOR* gene were found to occur at a higher frequency in patients with primary refractory AML and a mouse model with *BCOR* deletion showed increased resistance to the chemotherapeutic drug cytarabine, including by whole-exome sequencing (63). Another study found that loss-of-function mutations in *EZH2* can lead to the upregulation of several of its downstream target genes, which play a role in apoptosis evasion, proliferation enhancement, and altered function of membrane transporter proteins, thereby conferring a selective growth advantage on the cells and leading to chemoresistance (64).

## 4 Targeted therapies based on gene mutations

Although HMAs and cytotoxic chemotherapy have made some progress in the treatment of MDS, their efficacy is often limited by the heterogeneity of patients’ disease and drug resistance. HMAs have a low overall response rate and limited duration of remission, whereas conventional chemotherapy is more toxic and its use is limited in MDS patients of advanced age or with other comorbidities. Allo-HSCT is a potentially curative option, but is only available to a small number of young and well-conditioned patients and is at risk for graft-versus-host disease (GVHD) and post-transplant relapse (1, 2, 7, 9). With the deeper understanding of the complexity of the MDS genome and the emergence of novel targeted drugs, individualized therapeutic strategies based on genetic mutations will be the future direction of MDS treatment. By accurately characterizing the genome of a patient, we can select the most potentially effective targeted agents to improve response rates, prolong survival, and ultimately improve the clinical outcomes of MDS patients.

*TP53*, known as the “guardian of the genome,” is a 20-kb tumor suppressor gene located on chromosome 17p13.1. *TP53* encodes at least 15 different isozymes and has two paralogous relatives, p63 and p73, which share a similar structure and overlapping, but distinct, functions and upstream pathways (65). As a key tumor suppressor gene, the *TP53* gene is at the hub of a complex signaling network, which plays a central role in the maintenance of cell physiological functions by regulating the cell cycle, maintaining genome stability, and participating in key physiological processes such as cell metabolism, differentiation, proliferation, apoptosis, and senescence. In addition, *TP53* can activate downstream signals affecting the immune microenvironment and regulate immune-related targets, which in turn has a profound impact on the tumor microenvironment. The complexity and importance of its biological functions make it a key focus of cancer research (66–68). The frequency of *TP53* mutations is 5% ~ 10% in patients with *de novo* MDS and AML, and up to 20% ~ 40% in patients with elderly or treatment-related myeloid malignancies (69, 70). Most *TP53* mutations are missense substitutions and are concentrated in the DNA-binding domain, but there are multiple genetic aberrations of *TP53* in MDS and AML with complex functional consequences. The majority of *TP53-mutated* MDS patients with genomic and/or chromosome 17 abnormalities are “multi-hit,” with 24% with multiple mutations, 22% with deletion mutations, and 21% with copy number heterozygous deletions (66, 69, 70). *TP53* mutations, especially polygenic mutations, lead to poor clinical outcomes (36%). On the other hand, haploid *TP53* mutations (33%) frequently occur in combination with other genes, most commonly *TET2* (29%), *splicing factor 3b subunit 1(SF3B1)* (27%), *ASXL1* (16%), and *DNMT3A* (16%), and are likely to be late subclonal events, with varying prognostic implications (69, 71). Targeted therapies and immunotherapies for *TP53* mutations offer new hope for improving the prognosis of these patients (72).

**Figure 1:**
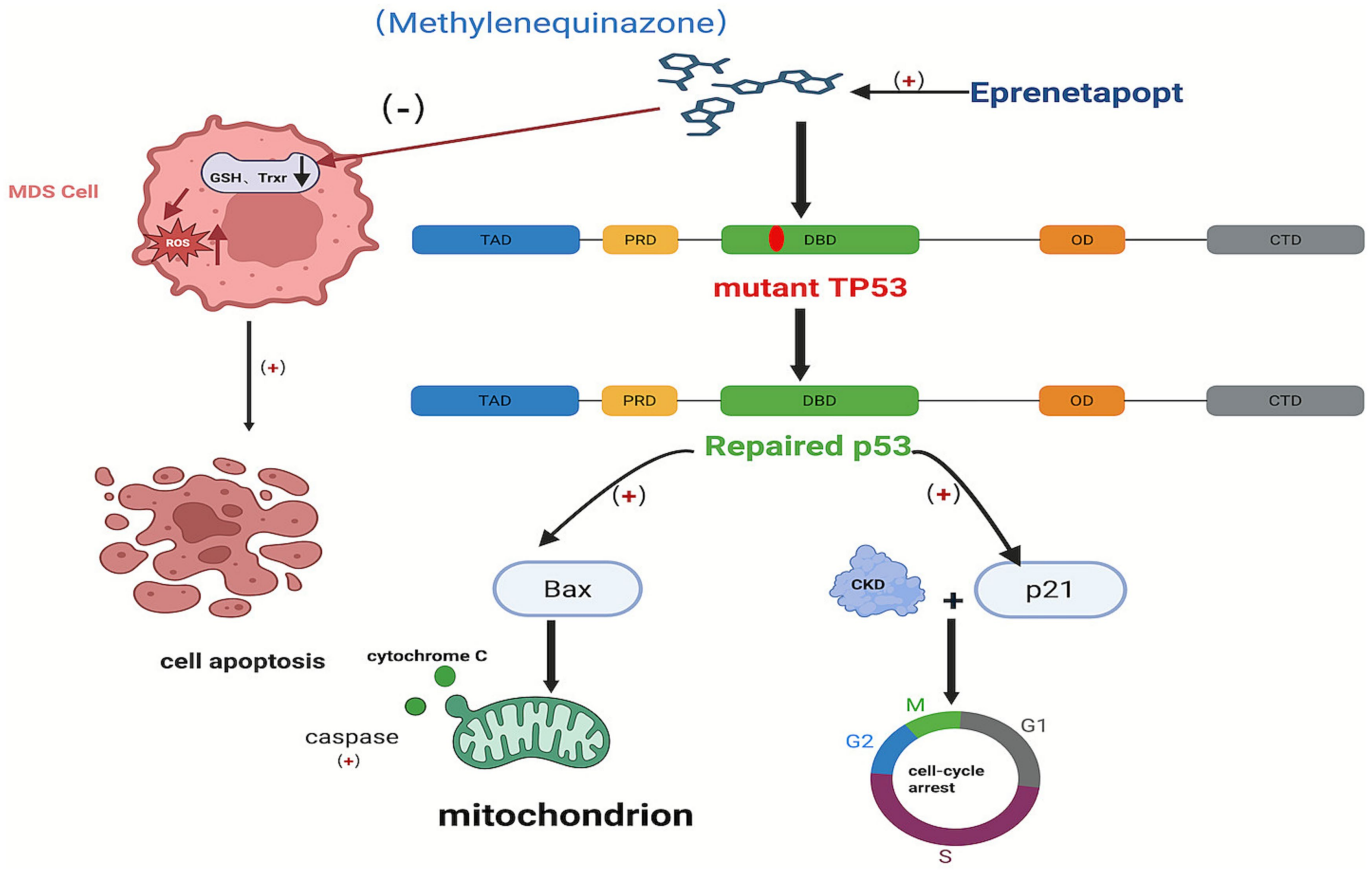
Mechanism of action of Eprenetapopt in the treatment of TP53-mutated MDS. This figure demonstrates the multiple mechanisms of Eprenetapopt (APR-246) in the treatment of MDS against TP53 mutations. Its active form, MQ, covalently binds to specific cysteine residues of TP53 mutant proteins, repairing the protein conformation and restoring its tumor suppressor function. The repaired p53 protein activates downstream p21 and Bax, with p21 blocking the cell cycle and Bax inducing apoptosis via the mitochondrial pathway. Meanwhile, Eprenetapopt depletes GSH, inhibits TrxR activity, and disturbs redox balance, resulting in ROS accumulation, leading to cell damage and apoptosis. Downstream p21 and Bax, with p21 blocking the cell cycle and Bax inducing apoptosis via the mitochondrial pathway. Meanwhile, Eprenetapopt depletes GSH, inhibits Tr activity, and disturbs redox balance, resulting in ROS accumulation, leading to cell damage and apoptosis. Created with BioRender.com.

The mechanism of action of Eprenetapopt (APR-246), a therapeutic agent for MDS targeting *TP53* mutations, is characterized by multiple aspects. First, Eprenetapopt enters the organism and is converted to the active form methylene quinazolone (MQ), which is able to covalently bind to specific cysteine residues in the core domain of *TP53* mutant proteins, such as Cys-124 and Cys-277 (73). This specific covalent binding alters the thermodynamic stability of the mutant p53 protein and restores its conformation to a near wild-type state, thereby reactivating its tumor suppressor function. The repaired p53 protein induces cell cycle arrest and promotes apoptosis by activating downstream target genes such as p21 and Bax. Among them, p21 binds to cell cycle protein-dependent kinase (CDK) and prevents cell cycle progression, while Bax induces cytochrome C release by altering mitochondrial permeability and activates the caspase cascade reaction, which contributes to apoptosis in MDS cells (37, 66, 74, 75). Secondly, Eprenetapopt also disrupts intracellular redox balance by depleting intracellular glutathione (GSH) and inhibiting the activity of thioredoxin reductase (TrxR), which is an important intracellular antioxidant, leading to a decrease in the intracellular antioxidant capacity, while the inhibition of TrxR affects a variety of Trx-dependent antioxidant defense mechanisms in the cell. Together, these two effects lead to the accumulation of reactive oxygen species (ROS) in MDS cells, causing lipid peroxidation, protein denaturation and DNA damage, and further inducing apoptosis in MDS cells. In addition, Eprenetapopt may synergize with drugs such as AZA. AZA, through its epigenetic mechanism, reactivates genes silenced by DNA methylation, thus enhancing the functional recovery of p53 mutant proteins by Eprenetapopt, and increasing the lethality of MDS cells (76–78) (Figure 1). A Phase Ib/II Eprenetapopt combined with AZA for the treatment of MDS and AML patients with *TP53* mutations clinical trial (NCT03072043) found that among 40 MDS patients, ORR was 73%, and CRR reached 50%; at the same time, the patients’ *TP53* variant allele frequency (VAF) and p53 protein expression were significantly reduced, and 38% of the patients achieved complete molecular remission (VAF < 5%), laying a solid foundation for subsequent phase III clinical trials, which is expected to change the current treatment landscape (78). Forty-nine patients with AML were enrolled in a multicenter phase I clinical trial (NCT04214860), which showed that VEN in combination with a HMA was well tolerated and resulted in an overall response rate of 38% when treated with Eprenetapopt and VEN in combination with AZA (79), but the subsequent phase III study was declared terminated due to failure to meet the prespecified endpoints (80). Another study found that Entospletinib in combination with DEC for the treatment of patients with *TP53-mutated* or CK-type AML showed only limited efficacy and was ultimately terminated from development due to a failure to achieve the desired effect (81). In addition, entrecitinib, an inhibitor targeting *ROS proto-oncogene 1 (ROS1)* receptor tyrosine kinase, has not yet achieved a substantial breakthrough in clinical application, although it has shown sustained sensitivity in *TP53*-deficient cells (82). Although clinical trials of these novel targeted agents are used in AML, this is also extremely informative for the diagnosis and treatment of MDS.

Mutations in *IDH1/2* lead to the accumulation of the oncogenic metabolite 2-hydroxyglutarate (2-HG), which affects cellular differentiation and leads to aberrant epigenetic regulation, which in turn blocks hematopoietic cell differentiation and promotes the progression of MDS to AML. The prevalence of *IDH2* mutations in MDS patients is approximately 2.1% ~ 4.0%, which is higher than that of *IDH1* mutations (0.6% ~ 3.6%). Enasidenib and Ivosidenib have been shown to be effective in MDS patients with *IDH1/2* mutations (83–85). A phase 1 multicenter, single-arm study (NCT02074839) found that ivosidenib-targeted therapy in patients with mutant *IDH1* (mIDH1) relapsed/refractory MDS (R/R-MDS) after failure of standard therapy resulted in a CRR of 38.9% and an ORR of 83.3% and a median overall survival (mOS) of 35.7 months. In terms of safety, 94.7% of patients experienced at least one treatment-related adverse event, of which 42.1% were treatment-related adverse events, most of which were grade 1–2, and no treatment-related deaths occurred (86). Marie et al. (87) found that Ivosidenib resulted in substantial hematologic improvement in MDS cohorts with different risk stratifications, particularly in first-treatment, high-risk patients, and was well tolerated overall, although the study was limited by a small sample size and a short follow-up period, and the results need to be further validated in larger, longer-term studies. Enasidenib acts primarily in mutant *IDH2 (mIDH2)* MDS. A subgroup analysis of the AG221-C-001 trial found that 17 patients with MDS harboring *mIDH2* were treated with Enasidenib with a median follow-up of 11.0 months. Results showed an ORR of 53% (95% CI 28–77%), a median duration of response of 9.2 months, a mOS of 16.9 months, and a median event-free survival of 11.0 months. The most common treatment-related adverse events were diarrhea and nausea (53% each), grade 3–4 adverse events included elevated indirect bilirubin (35%), pneumonia (29%), and thrombocytopenia (24%), and no treatment-related deaths occurred (88). DiNardo et al. (89) found that the Enasidenib monotherapy regimen in combination with AZA was efficacious and had a manageable safety profile in a phase II clinical trial of *mIDH2* MDS. Initial high-risk patients (Cohort A) had an ORR of 74%, a composite complete remission (CRc) rate of 70%, and a mOS of 26 months; patients who failed HMA therapy (Cohort B) had an ORR and CRc rate of 35% and a mOS of 20 months. Common adverse effects included neutropenia, nausea, constipation, and fatigue, while hyperbilirubinemia was observed in 14% of patients and *IDH* inhibitor-associated differentiation syndrome (IDH-DS) was observed in 16% of patients; however, the overall adverse effects were manageable, and no treatment-related deaths occurred. This study provides new therapeutic options for patients with high-risk *mIDH2* MDS, which is clinically important especially after failure of HMA therapy, and emphasizes the critical role of molecular marker testing in therapeutic decision-making.

## 5 Future perspectives

Current research on the prediction of MDS treatment response has made progress in some genes, but there are still many limitations. Taking HMAs treatment as an example, although it is clear that *ASXL1* mutation is associated with drug resistance and *TET2* mutation is associated with increased sensitivity, the accuracy and specificity of these predictive relationships still need to be improved, and some of the patients carrying *ASXL1* mutation are still effective in HMAs treatment, while some of the patients with *TET2* mutation are unresponsive to the treatment, which suggests that there are still other unknown genetic or non-genetic factors affecting treatment response. This indicates that there are other unknown genetic or non-genetic factors affecting the response to treatment, and future studies should deeply explore these potential factors, such as analyzing the effect of tumor cell heterogeneity on the response to treatment by single-cell sequencing, exploring the mechanism of mutations in non-coding regions other than coding regions and epigenetic modification changes on the sensitivity to HMAs, and constructing a more accurate prediction model by combining with bioinformatics analysis. For chemotherapy-related mutations, *RUNX1*, *BCOR*, and *EZH2* mutations are associated with poor chemotherapy efficacy, but their sensitivity and specificity in the prediction of chemotherapeutic response are also limited. In the future, it is necessary to further clarify the specific links between these mutations and the targets and mechanisms of chemotherapeutic drugs, and to explore the differences in their predictive value in different chemotherapeutic regimens. With the gradual introduction of targeted therapeutics against *TP53* and *IDH1/IDH2* mutations into clinical applications, the problem of drug resistance has become increasingly prominent. *TP53* targeted therapy, for example, has shown initial efficacy in some MDS patients with *TP53* mutations, but most of the patients will develop drug resistance after a period of treatment. It has been found that tumor cells can develop drug resistance through various mechanisms, so it is necessary to study the mechanism of drug resistance in depth in the future, and to overcome the drug resistance through the strategy of combining drugs, such as combining the use of *TP53* inhibitors with other drugs. For *IDH1/IDH2* inhibitors, some patients may experience disease progression during treatment. In the future, we should strengthen the monitoring and analysis of drug resistance-related mutations, develop new generation inhibitors, and explore the combination of other targeted drugs or epigenetic regulators. Regarding gene mutations and prognostic assessment, the existing prognostic scoring system is still not perfect in integrating gene mutation information. In the future, it is necessary to further optimize the prognostic scoring system to include more gene mutations with independent prognostic significance and dynamically adjust the prognostic assessment by combining the clinical characteristics of the patient and the response to treatment.
